# Effects of ADMA on gene expression and metabolism in serum-starved LoVo cells

**DOI:** 10.1038/srep25892

**Published:** 2016-05-16

**Authors:** Ningning Zheng, Ke Wang, Jiaojiao He, Yunping Qiu, Guoxiang Xie, Mingming Su, Wei Jia, Houkai Li

**Affiliations:** 1 Center for Chinese Medical Therapy and Systems Biology, Shanghai University of Traditional Chinese MedicineShanghai 201203, China.; 2 Laboratory of Integrative Medicine Surgery, Shuguang Hospital, Shanghai University of Traditional Chinese Medicine, Shanghai 201203, China; 3 Stable Isotope and Metabolomics Core Facility, Diabetes Center Albert Einstein College of Medicine, 1300 Morris Part Ave, Bronx, New York 10461, USA; 4 Cancer Epidemiology Program, University of Hawaii Cancer Center, Honolulu, Hawaii 96813, USA; 5 Center for Translational Medicine, and Shanghai Key Laboratory of Diabetes Mellitus, Department of Endocrinology and Metabolism, Shanghai Jiao Tong University Affiliated Sixth People’s Hospital, Shanghai 200233, China

## Abstract

Serum starvation is a typical way for inducing tumor cell apoptosis and stress. Asymmetric dimethylarginine (ADMA) is an endogenous metabolite. Our previous study reveals the plasma ADMA level is elevated in colon cancer patients, which can attenuate serum starvation-induced apoptosis in LoVo cells. In current study, we evaluated the effects of ADMA on gene expression and metabolism in serum-starved LoVo cells with gene microarray and metabolomic approaches. Our results indicated that 96 h serum starvation induced comprehensive alterations at transcriptional level, and most of them were restored by ADMA. The main signaling pathways induced by serum starvation included cancers-related pathways, pathways in cell death, apoptosis, and cell cycle etc. Meanwhile, the metabolomic data showed serum-starved cells were clearly separated with control cells, but not with ADMA-treated cells in PCA model. The identified differential metabolites indicated serum starvation significantly suppressed TCA cycle, altered glucose and fatty acids metabolism, as well as nucleic acids metabolism. However, very few differential metabolites were identified between ADMA and serum-starved cells. In summary, our current results indicated serum starvation profoundly altered the gene expression and metabolism of LoVo cells, whereas ADMA could restore most of the changes at transcriptional level, but not at metabolic level.

Asymmetric dimethylarginine (ADMA) is an endogenous inhibitor of nitric oxide synthase (NOS) derived from methylation of arginine residues in proteins by protein arginine methyltransferases (PRMTs). Elevated levels of ADMA in blood are typically observed in cardiovascular diseases, renal failure, pulmonary and hypertension, in which ADMA is regarded as an independent risk factor for cardiovascular diseases^1^,^2^,^3^. There are two types of PRMTs according to the specific catalytic activity. Type I (PRMT 1, 3, 4, 6 and 8) PRMTs catalyze the formation of ADMA, whereas type II (PRMT 5, 7 and FBXO11) produce symmetric dimethylarginine (SDMA)^4^,^5^. Study indicates that PRMT1 and 6 are significantly upregulated in various tumor tissues, as well as the increased level of their catalyzed product ADMA in blood of cancer patients. Meanwhile, the suppression of PRMT1 and 6 with siRNAs significantly inhibits the growth of bladder cancer cells (SW780 and RT4), and lung cancer cells (A549, LC319 and SBC5)^6^. These results suggest that the elevated ADMA may have biological function on tumor development. In another study, researchers find that the lower baseline levels of serum endothelin-1 and ADMA are correlated with the therapeutic responses to bevacizumab-based chemotherapy in metastatic colorectal cancer patients^7^, suggesting the probable association of ADMA with the responses upon anti-tumor chemotherapy. Recently, we observe that the plasma levels of ADMA in colon cancer patients are significantly higher than healthy subjects. Moreover, we find that ADMA can attenuate serum starvation-induced cell death and apoptosis on a colon cancer cell line LoVo cells, as well as protect LoVo cells from doxorubicin hydrochloride-induced cell death^8^.

Despite the novel findings of elevated ADMA in colon cancer patients, as well as its anti-apoptosis effect in serum-starved LoVo cells, the mechanism underlying protection against serum starvation-induced apoptosis of ADMA in LoVo cells is still of little known so far. In order to understand the global impacts of ADMA on gene expression and metabolism on LoVo cells, we first performed transcriptomic profiling of ADMA in serum-starved LoVo cells using gene expression microarray. Given the observed anti-apoptosis effect of ADMA in LoVo cells induced by serum starvation, then, the expression of genes in cell apoptosis and death pathway was further validated with RT^2^ Profiler PCR Arrays. Meanwhile, an untargeted metabolic profiling was conducted based on GC/TOFMS and LC/TOFMS metabolomic platforms. Our current results indicated that serum starvation could result in comprehensive changes both at transcriptional and metabolic levels, while ADMA treatment significantly restored the transcriptional alterations induced by serum starvation, especially the genes in cell apoptosis and death pathway, but showed minor impacts on LoVo cell metabolism.

## Experimental Section

### Cell culture and treatment

Human colon cancer LoVo cells (CCL-229) from ATCC were cultured in 10 cm dishes at 37 °C in a humidified atmosphere of 5% CO_2_ in 10% FBS DMEM supplemented with 100 U/ml penicillin and 100 mg/ml streptomycin. Cells were cultured in normal culture medium (10% FBS DMEM), serum starvation medium (0% FBS DMEM) and 10 μM ADMA (0% FBS DMEM) for 96 h without addition of any growth factors. Then, the transcriptomic and untargeted metabolic profiling were conducted with the samples of RNA and extraction of cellular metabolites, respectively.

### Gene expression analysis with microarray

Cells were collected and total RNA was extracted using TRIzol reagent (Invitrogen, Carlsbad, CA), followed by purification on an RNeasy column (Qiagen, Germantown, MD) and quantified by UV absorption (Nanodrop, Thermo Scientific). RNA quality was evaluated by RNA 6000 Nano LabChip (Agilent Technologies, Santa Clara, CA) on an Agilent 2100 Bioanalyzer. Following quantification, 1 μg of each total RNA sample was amplified, labeled and hybridized according to the standard Affymetrix protocols by Expression Analysis, Inc. (Durham, NC, USA). The platform was used the Affymetrix HG-U133_Plus_2.0 Array. Expression values were calculated using Affymetrix GeneChip analysis software MAS 5.0. CEL files have been deposited to the Gene Expression Omnibus database (http://www.ncbi.nlm.nih.gov/geo) with the accession number GSE70976.

### Metabolomic analysis

The metabolomic analysis of cellular metabolite extraction was carried out with GC/TOFMS (Pegasus HT system, Leco Corporation) and LC/TOFMS (Agilent Corporation) after the two-step extraction of cellular metabolites with mixed solvent. The cellular metabolite extraction process was described as following: Place the 10 cm dishes containing about 1 × 10^7^ cells/each on ice. Remove the culture medium and wash with 5 ml of ice-cold isotonic saline (0.9% [w/v] NaCl for three times. Add 1 ml of ice-cold 0.9% NaCl and scrape the cells off carefully. Transfer the cells into a 1.5 ml centrifuge tube, and centrifuge at 1000 × g for 2 min at 4 °C. Remove the supernatant and repeat the washing step twice with 0.9% NaCl. Before the metabolites extraction, a 10 μl of cell suspensions were allocated into another tube for protein quantification. The total protein was extracted using the Mammalian Protein Extraction reagent (Thermo Scientific, Rockford, IL, USA), and determined with the Pierce Coomassie Protein Assay Kit (Thermo Scientific) for the subsequent normalization of metabolic data. The harvested cells were added with 350 μl of methanol : chloroform : water (2.5:1:1 [v/v/v]) mixed solvent which has been refrigerated at −20 °C completely. Vortex for 30 s and sonicate for 5 min in ice water. Centrifuge at 13,200 rpm for 15 min at 4 °C. Transfer 175 μl of supernatant to a GC and LC sampling vial respectively for the following GC/TOFMS and LC/TOFMS analysis. The sample residue was extracted with 350 μl of methanol again. Vortex for 30 s, and sonicate for 5 min. Centrifuge at 13,200 rpm for 15 min at 4 °C, and transfer 175 μl of supernatant to the GC and LC sampling vials respectively. Then, each sample was added with internal standards (10 μL heptadecanoic acid at 1 mg/mL and 4-chlorophenylalanine at 0.3 mg/mL). For GC/TOFMS analysis, the metabolite extraction was vacuum dried at room temperature, and the residue was then chemically derivatized with BSTFA, while the LC/TOFMS was performed directly with the solvent extraction. The protocols for GC/TOFMS and LC/TOFMS were described previously^9^,^10^.

### Validation of genes in cell death pathway with RT^2^ Profiler PCR Array

Following the analysis of transcriptomic data, commercial RT^2^ Profiler PCR Arrays were used for quantitation of 84 targeted genes involved in cell death pathway (Cat no. PAHS-212Z, QIAGEN, Germany) in LoVo cells treated with or without 10 μM ADMA for 48 and 96 h, which are independently different with those used for transcriptomic profiling. Briefly, the total RNA was extracted with RNeasy Mini Kit (Cat no. 74104, QIAGEN, Germany) and 1 μg of total RNA was subjected to first strand cDNA synthesis with RT^2^ HT First Strand Kit (Cat no. 330411, QIAGEN, Germany) according to the manufacturer’s instructions. The synthesized cDNA samples were analyzed with RT^2^ Profiler PCR Arrays in triplicate on ABI7500 system (ABI).

### Data analysis

The data analysis of both GC/TOFMS and LC/TOFMS were described in our previous publications^11^,^12^. First of all, the generated GC/TOFMS data were normalized to internal standard and the total protein of each sample. Then, the normalized GC/TOFMS data were analyzed in the SIMCA-p 11.0 Software package (Umetrics, Umeå, Sweden). The unsupervised multivariate statistic, principal component analysis (PCA) was first used to compare the metabolic profiles between groups. Differential variables were then selected with the criteria of variable importance in the projection (VIP > 1) in the partial least-squares-discriminant analysis (PLS-DA) model and p < 0.05 in a Student’s t-test. Compound identification was performed by comparing the mass fragments of interesting variables with NIST 05 standard mass spectral databases in NIST MS search 2.0 (NIST, Gaithersburg, MD) software at a similarity score of greater than 70%. The LC/TOFMS data were analyzed with MassHunter Qualitative Analysis Program (vB.03.01) (Agilent), and the XCMS package (v1.24.1) (http://metlin.scripps.edu), which runs in the statistical package R (v.2.12.1) (http://www.r-project.org), to pick, align, and quantify features (chromatographic events corresponding to specific m/z values and rention times). The generated raw data were normalized to internal standard and total protein of each sample for further analysis. Metabolite annotation was performed by comparing the accurate mass (m/z) and retention time (Rt) of reference standards in our in-house library and the accurate mass of compounds obtained from the web-based resources such as the Human Metabolome Database (http://www.hmdb.ca/) and The METLIN Metabolite Database (http://metlin.scripps.edu/).

For the microarray data analysis, only those probes with “perfect value” present in >2 samples (60%) in each group were applied in further analysis. To visualize the general differences of gene expression among groups, an unsupervised PCA was performed by using the entire gene expression dataset, in which all the probes have been filtered with R version 3.0.1. The significance analysis of microarrays (SAM) method was used for evaluation of changes in gene expression at the false discovery rate (FDR) < 0.01 and cutoff of 1.2-fold changes^13^. Gene Ontology (GO) enrichment analysis of the differentially expressed genes was performed using the database for annotation, visualization, and integrated discovery (DAVID) (http://david.abcc.ncifcrf.gov/)^14^. To identify significant enrichment of GO terms, the expression analysis systematic explorer (EASE) score threshold in DAVID was set at ≥1.3 (p < 0.05). Meanwhile, to provide a functional outline for function interpretation, regulated gene pathways were explored by using Kyoto Encyclopedia of Genes and Genomes (KEGG) online database (http://www.genome.jp/kegg/) by DAVID^15^. Hierarchical clustered heat maps were produced with Cluster 3.0 and TreeView software (M. B. Eisen Laboratory, Stanford University, Stanford, CA).

## Results

### Serum starvation resulted in global transcriptional alteration on LoVo cells

**Figure 1:**
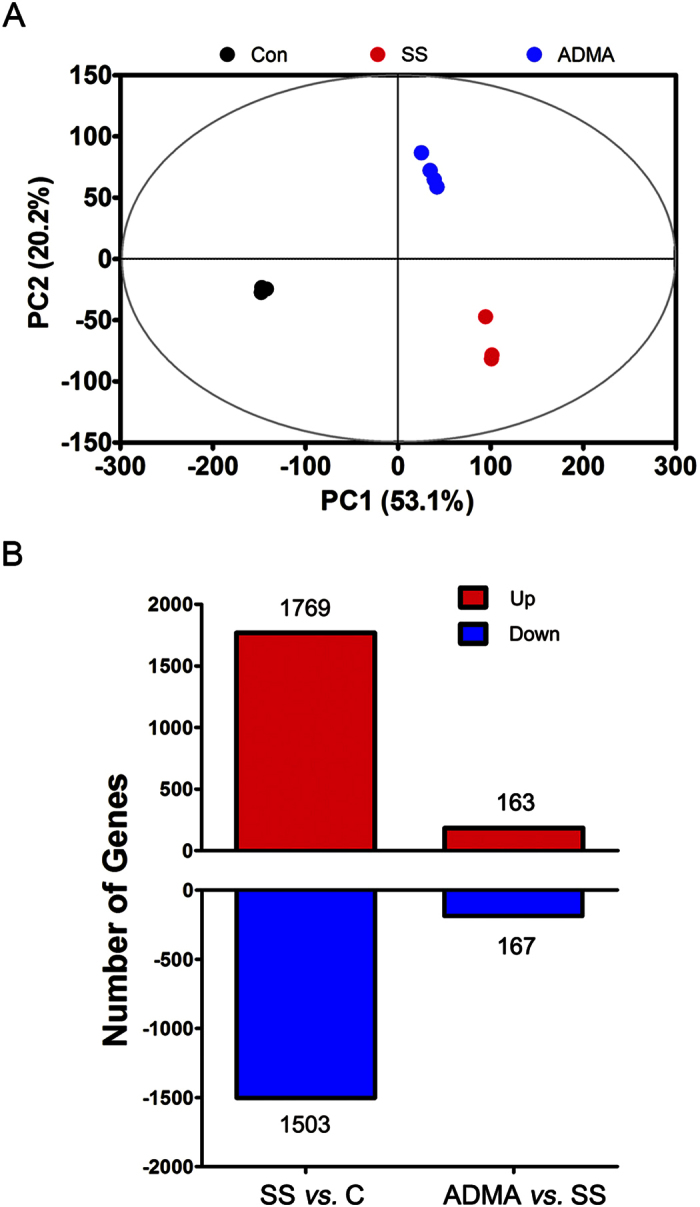
Effects of serum starvation and ADMA treatment on the transcriptome of LoVo cells. (**A**) The PCA score plot of transcriptomic profiles of three groups. (**B**) The numbers of differentially expressed genes by serum starvation *vs.* control, and ADMA *vs.* serum starvation. C: control group; SS: 96 h serum starvation group; ADMA: 10 μM ADMA for 96 h in serum starvation medium.

Previously, our results indicate that 96 h serum starvation significantly reduces cell viability and increases cell apoptosis in LoVo cells^8^. To further understand the globally transcriptional impacts of serum starvation on LoVo cells, we first performed transcriptomic profiling with all the detected genes in control and 96 h serum-starved cells. An unsupervised PCA model was constructed which showed a distinct separation between control and serum-starved cells (Fig. 1A), suggesting the dramatic transcriptional impacts of serum starvation on LoVo cells.

**Figure 2:**
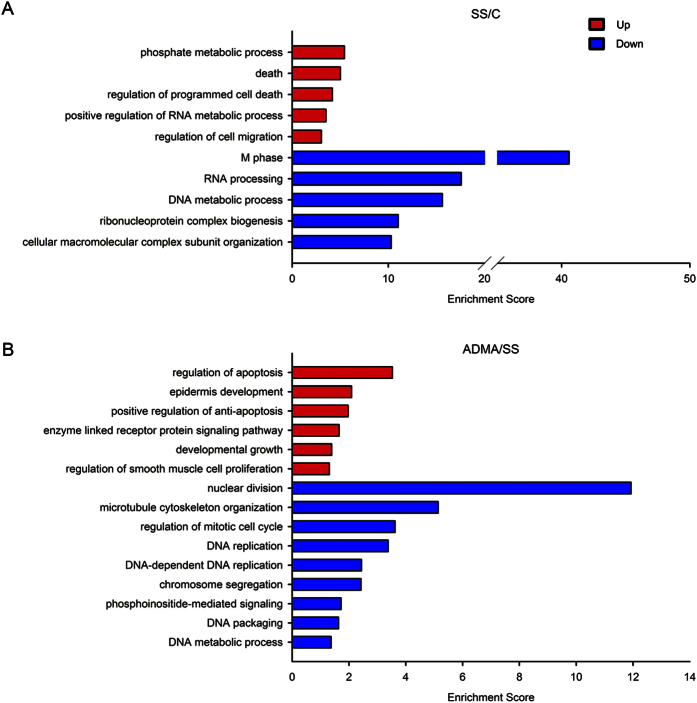
Enriched GO terms by serum starvation and/or ADMA treatment for 96 h in LoVo cells. (**A**) The top 5 enriched GO terms in up- or down-regulated genes by serum starvation treatment. (**B**) The all enriched GO terms in up- and down-regulated genes by ADMA treatment compared to serum starvation treatment. C: control group; SS: 96 h serum starvation group; ADMA: 10 μM ADMA for 96 h in serum starvation medium.

Moreover, the microarray results showed there were 1769 and 1503 genes were significantly up- and down-regulated respectively by serum starvation (Fig. 1B). To obtain insight into the broader functional roles of these serum starvation-regulated genes, we performed their enrichment in GO terms associated with biological processes. The Top 5 enriched GO terms within up- and down-regulated genes were present in order of DAVID Enrichment score (Fig. 2, see complete enriched GO terms in Supplementary Table S1). These results highlighted functional involvement of serum starvation-associated genes in cell death and viability (*death*, *regulation of programmed cell death*, *regulation of cell migration*), cellular metabolic process (phosphate metabolic process, *positive regulation of RNA metabolic process*, *RNA processing*, *DNA metabolic process*, *ribonucleoprotein complex biogenesis*, and cellular macromolecular complex subunit organization), and cell cycle (*M phase*). In addition, to better understand higher-order functional association, we examined gene enrichment in KEGG pathway. With the pathway enrichment analysis, 11 pathways were enriched, which included the *cell cycle pathway*, *p53 signaling pathway*, regulation of transcription (*DNA replication*, *spliceosome*, *base excision repair*, *nucleotide excision repair*, *mismatch repair*), and pathways involved in nucleic acid metabolism (*pyrimidine metabolism*, *pyrimidine metabolism*, *glutathione metabolism*, *purine metabolism*, *RNA degradation*) (Table 1). Altogether, the gene expression profiling indicated that serum starvation induced comprehensive transcriptional alterations on LoVo cells, which are mainly involved in cell cycle, apoptosis, nucleic acids metabolism and p53 signaling pathways.

### ADMA attenuated serum starvation-induced transcriptional alteration

**Figure 3:**
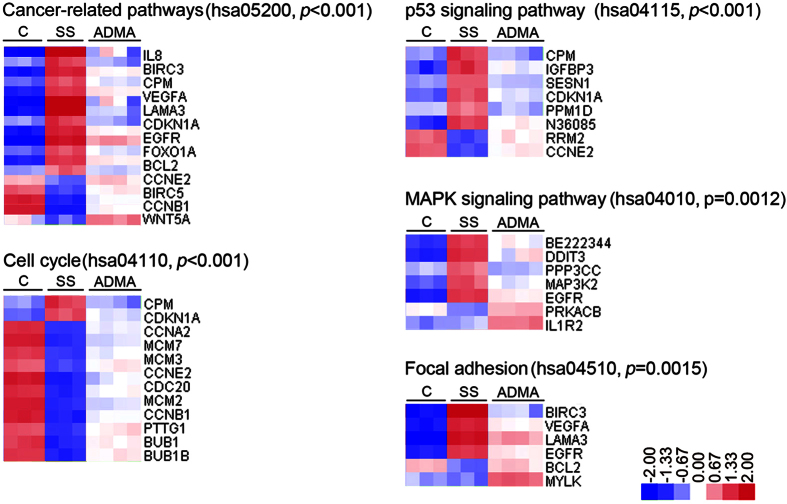
Enriched KEGG pathways and regulated genes by serum starvation and/or ADMA treatment for 96 h in LoVo cells. Log2 ratios were color coded as indicated. C: control group; SS: 96 h serum starvation group; ADMA: 10 μM ADMA for 96 h in serum starvation medium.

In our previous report, we demonstrate that 10 μM ADMA treatment attenuates serum starvation-induced cell viability reduction and apoptosis on LoVo cells^8^. To test whether ADMA treatment could attenuate the serum starvation-induced transcriptional changes on LoVo cells, the LoVo cells were treated with 10 μM ADMA simultaneously in serum-starved medium for consecutive 96 h. First of all, the PCA model of global transcriptional profiling showed that the gene expression pattern of ADMA-treated cells was clearly separated from both control and serum-starved cells (Fig. 1A), suggesting the unique gene expression pattern of ADMA-treated cells. To further characterize the transcriptional impacts of ADMA on serum-starved cells, we compared the gene microarray data from ADMA and serum-starved groups directly. There were 163 and 167 up- and down-regulated genes between ADMA-treated and serum-starved cells (Fig. 1B; Supplementary Table S2 and Figure S1). Interestingly, the expression levels of most ADMA-regulated genes were between control and serum-starved groups, suggesting that the ADMA treatment could attenuate the effect of serum starvation (Figure S1). Gene-enrichment analyses identified several distinct processes that were significantly associated with the up- and down-regulated genes (Fig. 2). For genes down-regulated, the associated enriched GO terms included apoptosis (*regulation of apoptosis*, *positive regulation of anti-apoptosis*), cellular development (*epidermis development*, *developmental growth*, *regulation of smooth muscle cell proliferation*), and signaling pathway (*enzyme linked receptor protein signaling pathway*), whereas the up-regulated genes included regulation of transcription (*nuclear division*, *regulation of mitotic cell cycle*, *DNA replication*, *DNA-dependent DNA replication*, *chromosome segregation*, *DNA packaging*, *DNA metabolic process*) and signaling pathway (*phosphoinositide-mediated signaling*). Meanwhile, the enriched KEGG pathways in serum-starved cells were mainly involved in *cancer-related pathways*, *p53 signaling pathway*, *MAPK signaling pathway*, *focal adhesion*, and *cell cycle,* whereas most of the transcriptional changes were reversed by ADMA treatment (Fig. 3).

### Validation of genes in cell death pathway with qRT-PCR

**Figure 4:**
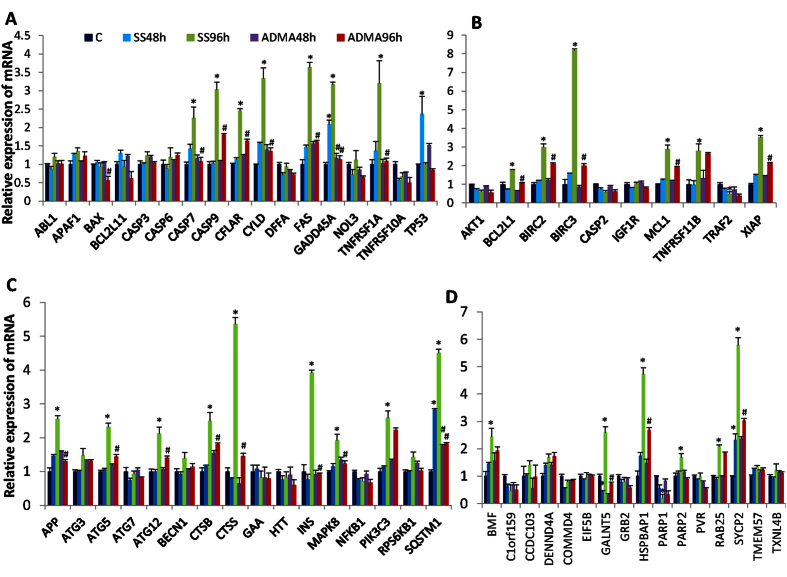
The expression analysis of genes involved in cell death pathways by using RT^2^ Profiler Array. (**A**) Genes in pro-apoptosis pathway; (**B**) Genes in anti-apoptosis pathway; (**C**) Genes in autophagy pathway; (**D**) Genes in necrosis pathway; Data are means ± S.E.M. of triplicates at least for each group. *indicates P < 0.05 compared to control group, ^#^P < 0.05 compared to the corresponding serum starvation group with Student’s *t* test.

Since ADMA treatment could attenuate serum starvation-induced cell death, and restore most of the transcriptional changes of serum starvation, we further validated the expression of 84 genes in cell death pathways in ADMA-treated cells after either 48 or 96 hours with qRT-PCR analysis for characterization of the time-dependent transcriptional effects. The analyzed genes are involved in pathways of pro-, and anti-apoptosis, autophagy and necrosis. Among the 84 genes, 25 genes were excluded from the final analysis because of extremely low presence in all samples. In general, 96 h serum starvation resulted in an obvious up-regulation in most of the observed genes, whereas only several genes were significantly changed by 48 h serum starvation (Fig. 4). In pro-apoptosis pathway, eight out of 18 observed genes were dramatically up-regulated by 96 h serum starvation including Baculoviral IAP repeat containing 2 (BIRC2), apoptosis-related cysteine peptidase (CASP7), CASP9, CASP8 and FADD-like apoptosis regulator (CFLAR), cylindromatosis (CYLD), TNF receptor superfamily, member 6 (FAS), growth arrest and DNA-damage-inducible, alpha (GADD45A), and tumor necrosis factor receptor superfamily, member 1A (TNFRSF1A), and all these up-regulated genes were down-regulated by 96 h ADMA treatment (Fig. 4A). On the other hand, 96 h serum starvation also stimulated the expression of several genes in anti-apoptotic pathway, such as BCL2-like 1(BCL2L1), Baculoviral IAP repeat containing 3 (BIRC3), Myeloid cell leukemia sequence 1 (MCL1), Tumor necrosis factor receptor superfamily, member 11b (TNFRSF11B), and X-linked inhibitor of apoptosis (XIAP), in which only BIRC3 and XIAP were down-regulated by ADMA treatment (Fig. 4B). Autophagy is paradoxical in tumor development, which has been reported to have roles in either promoting tumor cell survival or cell death^16^. We observed that most of the observed genes in regulation of autophagy were up-regulated by 96 h serum starvation, and down-regulated by 96 h ADMA treatment (Fig. 4C). Sequestosome 1 (SQSTM1) encodes a multifunctional protein that plays important roles in regulation of signaling pathway of inflammation and autophagy. We observed that SQSTM1 was time-dependently stimulated by serum starvation, and significantly suppressed by ADMA treatment at either 48 or 96 h (Fig. 4C). In addition, several genes in regulation of necrosis process were also greatly up-regulated by 96 h serum starvation such as Bcl2 modifying factor (BMF), polypeptide N-acetylgalactosaminyltransferase 5 (GLANT5), HSPB (heat shock 27kDa) associated protein 1 (HSPBAP1), poly (ADP-ribose) polymerase 2 (PARP2), RAB25, and synaptonemal complex protein 2 (SYCP2) (Fig. 4D). Altogether, our current data of genes in cell death pathways indicated that 96 h serum starvation stimulated the expression of most of the genes involved in regulation of cell apoptosis, autophagy and necrosis process, which could be attenuated by simultaneous treatment of ADMA in LoVo cells.

### The global metabolic influences of serum starvation and ADMA on LoVo cells

**Figure 5:**
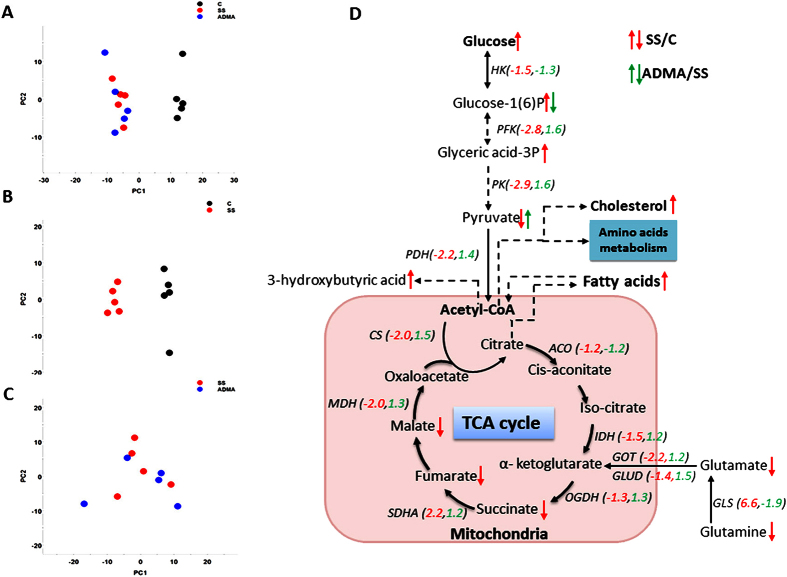
Metabolic profiling of LoVo cells treated with or without 10 μM ADMA for 96 h in serum- starved medium. (**A–C**) The PCA score plots for all three groups, C vs SS, and SS vs ADMA, respectively. (**D**) Summary of main differential metabolites and transcriptional changes of corresponding genes altered by serum starvation and ADMA in glucose metabolism and TCA cycle. The dashed arrows in metabolic processes represent reactions with multiple steps. The red and green Arabic numerals represent the transcriptional fold change of the genes of SS/C, as well as ADMA/SS, respectively. C: control group; SS: 96 h serum starvation group; ADMA: 10 μM ADMA for 96 h in serum starvation medium. HK: hexokinase; PFK: phosphofructokinase; PK: pyruvate kinase; PDH: pyruvate dehydrogenase; ACO: aconitase; IDH: isocitrate dehydrogenase; OGDH: α-ketoglutarate dehydrogenase; SDHA: succinate dehydrogenase; MDH: malate dehydrogenase; CS: citrate synthase; GOT: glutamate oxaloacetate transaminase; GLUD: glutamate dehydrogenase; GLS: glutaminase.

To evaluate the global metabolic impacts of serum starvation, we performed unsupervised PCA with the identified 221 metabolites from both GC/TOFMS and LC/TOFMS. The PCA loading plots showed that the samples from both serum-starved and ADMA-treated groups were distinctly separated from control group, while samples between serum-starved and ADMA-treated cells clustered together (Fig. 5A–C), suggesting the serum starvation-induced metabolic changes could not be completely restored by ADMA treatment. To further characterize the metabolic impacts of serum starvation on LoVo cells, as well as the metabolic effects of ADMA on serum-starved cells, we used supervised PLS-DA models to differentiate the groups between control and serum starvation, serum starvation and ADMA treatment. A total of 57 differential metabolites were identified between serum starvation and control cells. About 2/3 of the differential metabolites were up-regulated by serum starvation (Table 2). These differential metabolites were mostly involved in the energy metabolism pathways and nucleic acids metabolism. We observed that many metabolites in glucose metabolism pathways were increased by serum starvation such as glucose, glucose 1(6)-phosphate, myo-inositol phosphate, 3-hydroxybutyric acid, glycerol 3-phosphate, glyceric acid, 3-phosphoglyceric acid, and glucuronic acid, as well as several other monosaccharides including fructose, mannose, galactose, xylose, and arabinose, whereas metabolites in TCA cycle were reduced by serum starvation such as succinic acid, fumaric acid, and malic acid. Serum starvation increased cellular contents of fatty acids such as pentadecanoic acid (15:0), palmitoleic acid (16:1), oleic acid (18:1), eicosenoic acid (20:1) and eicosapentaenoic acid (20:5). Meanwhile, serum starvation also elevated the levels of metabolites in nucleic acids metabolism pathway such as deoxyguanosine, uracil, inosine, adenosine, and guanosine, except for the decreasing of adenine. In addition, serum starvation resulted in the alterations of amino acids and derivatives of amino acids including the increasing of proline, glycine, methionine, lysine, homocysteine and decreasing of asparagine, aspartic acid, aminoadipic acid 5-aminolevulinic acid, 5-oxoproline, 4-hydroxy-proline, glutathione, glutamate, and glutamine. Although ADMA could exert significant impacts on serum-starved cells at transcriptional level, the metabolic effects were minor. Among the 57 serum starvation-induced differential metabolites, only 3 metabolites were conversely regulated by ADMA, which are pyruvic acid, fructose and glucose 1(6)-phosphate. To integrate the metabolic and transcriptional results, we further screened the transcriptional changes of genes that catalyze the identified metabolic pathways from microarray data. We found that the majority of genes were down-regulated by serum starvation in glycolysis and TCA cycle, while most of them were at least partially reversed by ADMA treatment. On the contrary, the expression of glutaminase (GLS) gene, which catalyzes the conversion of glutamine to glutamate, was dramatically stimulated by serum starvation, and suppressed by ADMA. The altered main metabolic pathways and changes of the corresponding genes were summarized in Fig. 5D.

## Discussion

ADMA is an endogenous NOS inhibitor which is elevated in cardiovascular disease^17^ and cancers^6^. However, the biological functions of ADMA in tumor cells are unclear. We previously demonstrated that serum starvation triggered the apoptosis on LoVo cells, which was attenuated by ADMA treatment^8^. Smith *et al*. treat the human coronary artery endothelial cells with pathophysiological concentrations of ADMA and observe the global effects on gene expression^18^. They find that the pathophysiological concentrations of ADMA elicit significant changes in gene expression and interestingly some of the changes are independent of L-arginine: NO pathway suggesting that the biological functions of ADMA are more complicated than its typical role as NOS inhibitor. In current report, our results indicated that 96 h serum starvation could induce comprehensive transcriptional and metabolic alterations in LoVo cells, whereas ADMA could effectively restore most of the transcriptional changes, but had minor impacts on cell metabolism.

Serum starvation is a typical way for reducing cell viability and triggering cell apoptosis^8^,^19^. However, the mechanisms underlying serum starvation-induced apoptosis are complicated and vary across different cell lines and under different starvation conditions^20^. In some cell lines such as undifferentiated PC-12 cells^21^, mouse fibroblasts^22^, and embryo cells^23^, a rapid apoptosis could be observed within several hours, whereas apoptosis usually takes place after a few days of serum starvation in HUVEC^24^, differentiated PC-12 cells^21^, as well as LoVo cells^25^. Consistently, we observed a significant increase in apoptosis rate after 96 h serum starvation in LoVo cells^8^. There are few reports in respect to the global gene expression profile upon serum starvation in tumor cells. In our study, we found serum starvation resulted in over 3000 differently expressed genes, in which the top 5 clusters of up- and down-regulated signaling pathways were mainly involved in cell death, nucleic acid metabolism and cell cycle regulation. The following KEGG pathway enrichment analysis identified 11 signaling pathways that were significantly altered by 96 h serum starvation including mainly cell cycle, p53 pathway, nucleic acid metabolism and glutathione metabolism. We observed the obvious up-regulation of apoptosis process by serum starvation, and anti-apoptosis signaling pathway by ADMA, which is consistent with their phenotypic effects on apoptosis^8^.

TP53 (p53 gene) is a tumor suppressor gene and plays important roles in regulation of apoptosis, genomic stability, and cell cycle^26^. In our current report, we found that serum starvation significantly stimulated the expression of p53 gene at 48 h, but not at 96 h compared to control group. Given the observed apoptosis only at 96 h of serum starvation in our study^8^, it is suggested that p53 gene is not necessary for serum starvation-induced apoptosis in LoVo cells, which is consistent with the previous results that serum starvation-induced apoptosis is p53 independent in LoVo cells^25^. Fas (APO-1/CD95) is an important member of TNF receptor superfamily^27^ and plays crucial role in modulating immune function and apoptosis^28^. Our current result indicated that the expression of Fas gene was up-regulated by serum starvation at 96 h and suppressed by ADMA treatment, which was consistent with our previous observation^8^. In primary granulosa cells, both p53 and Fas pathways are significantly stimulated by 2 days serum starvation^29^. Accordingly, the p53 gene may play different roles in apoptosis process induced by serum starvation in different cells. Moreover, given the role of post-translational modification of p53 and the effects of activated p53 on regulation of cell cycling and apoptosis, our current results could not determine whether the anti-apoptosis effect of ADMA was p53-dependent or independent.

Serum starvation-induced apoptosis is usually caspase-dependent^30^. Caspases 3, 6, and 7 are classified as executioner caspases, which cleave the protein substrates within the cell and initiate the apoptotic process. Caspases 2, 8, 9, and 10 are initiator caspases that cleave inactive pro-forms of executioner caspases leading to their activation. It is envisaged that the caspase-dependent apoptotic process is cell line-specific. In rat 423-cells, serum starvation initiates time-dependent apoptosis in 24 h, and the caspases 8 and 3 are cleaved within the early 6 h, whereas caspase 9 remains unaffected^30^. In our study, we observed that the expression of caspases 7 and 9 were up-regulated by serum starvation at 96 h, whereas caspases 2, 3 and 6 were not affected within 96 h serum starvation, suggesting that serum starvation-induced apoptosis is probably associated with activation of caspases 7 and 9. Accordingly, the regulation of caspases in serum starvation-induced apoptosis is a complex process, which can be influenced by cell type and culture system^31^. Growth arrest and DNA-damage-inducible, alpha (GADD45A) is a member of a group of genes that are stimulated upon stresses and DNA-damaging agents. The pro-apoptotic activity of GADD45A protein has positioned it as an essential player in tumorigenesis^32^. GADD45A is up-regulated upon serum starvation in intervertebral disc cells^33^. We observed that serum starvation could time-dependently stimulate the expression of GADD45A in LoVo cells, which was suppressed by ADMA treatment at both 48 and 96 h. It is reported that the expression of GADD45A gene is associated with lymph node metastasis, tumor stage and tumor histological grade in patients with oral squamous cell carcinoma^34^. Given the important role of GADD45A in modulating cell responses to DNA damage and association with tumor prognosis, GADD45A is probably involved in ADMA-mediated signaling pathways in tumor cells.

In addition, we also observed several genes with anti-apoptotic functions were significantly stimulated by 96 h serum starvation, and most of them were down-regulated by ADMA such as baculoviral IAP repeat containing 2 (BIRC2), BIRC3, myeloid cell leukemia 1 (MCL1), TNFRSF11B and X-linked inhibitor of apoptosis, E3 ubiquitin protein ligase (XIAP). Therefore, these changes of genes in either pro-apoptotic or anti-apoptotic pathways highlighted that long term serum starvation not only gave rise to apoptosis, but also initiated the anti-apoptosis responses in LoVo cells to survive the microenvironment of nutrients deprivation.

The long term nutrients deprivation could produce multi-facets impacts on tumor cells including the nutrients deprivation-induced apoptosis, and adaptive changes for survival such as autophagy^35^. In current study, we found that 9 out of 16 autophagy-related genes were significantly stimulated by 96 h serum starvation, and most of them were down-regulated by simultaneous ADMA treatment. Sequestosome 1(SQSTM1) gene encodes a multifunctional protein, which serves as a scaffolding hub in various cellular signaling pathways such as NF-κB activation and caspase activation^36^. SQSTM1 plays important roles in various diseases such as obesity, tumors, and bone metabolism^37^,^38^, and is rapidly degraded at the early stage of amino acids and serum starvation-induced autophagy^39^, but restored during prolonged starvation in mouse embryonic fibroblasts and HepG2 cells^35^. In our current study, we observed that the expression of SQSTM1 gene was time-dependently up-regulated by 48 and 96 h serum starvation, but suppressed by ADMA simultaneously in LoVo cells. It is hypothesized that the restoration of SQSTM1 in nutrients starvation is associated with the availability of autophagy-derived amino acids^35^. Meanwhile, several other autophagy-related genes were also up-regulated by 96 h serum starvation and inhibited by ADMA treatment such as amyloid beta (A4) precursor protein (APP), autophagy related 5 (ATG5), ATG12, cathepsin B (CTSB), cathepsin S(CTSS), and mitogen-activated protein kinase 8 (MAPK8). Since tumor cells can survive in serum starvation or metabolic stress by triggering autophagy^40^, it was suggested that a simultaneous autophagy process might be initiated by serum starvation, in addition to the apoptosis process in LoVo cells, and ADMA treatment could reverse the transcriptional changes of genes in autophagy, but further investigation is needed to examine whether the autophagy was presence in serum-starved cells and inhibited by ADMA treatment.

Serum starvation is a potent cellular stress which can induce comprehensive impacts on cell metabolism. However, to the best of our knowledge, there are few reports on the global metabolic impacts of serum starvation on tumor cells so far. In current study, we observed serum starvation induced comprehensive metabolic impacts on LoVo cells, leading to over 50 differential metabolites that are mainly involved cell energy metabolism pathways such as glucose metabolism, TCA cycle, fatty acids metabolism, amino acids metabolism, and nucleic acid metabolism. Over half of the metabolites were up-regulated in serum starved-cells, especially metabolites involved in glucose, fatty acid and nucleic acids metabolism, whereas some metabolites in TCA cycle and amino acids were greatly down-regulated. It is well-established that serum starvation is an effective initiator for apoptosis in cells^30^, which is directly associated with the mitochondrial damage and glutathione oxidation^41^. In LoVo cells, we found that the cellular content of glutathione was completely depleted by serum starvation, as well as over five folds increase of homocysteine. Homocysteine is an inducer of oxidative stress and cellular apoptosis, which plays important role in cardiovascular diseases and cancers^42^,^43^,^44^. The up-regulation of cellular homocysteine and depletion of glutathione indicated that the serum starvation-induced mitochondrial damage, as well as apoptosis.

In tumor cells, both glucose and glutamine are important nutrients for tumor cell proliferation through aerobic glycolysis and glutaminolysis^45^. The deprivation of glucose and glutamine for several hours will lead to the reduction of cell numbers in HeLa cells^46^. In current study, we found that serum starvation resulted in dramatic depletion of glutamine and glutamate, but significant accumulation in glucose and metabolites in glycolysis process such as glycerol-3-phosphate, glyceric acid, 3-phosphoglyceric acid, and glucose 1(6)-phosphate, as well as the reduction of metabolites in TCA cycle such as succinic acid, fumaric acid, and malic acid. Since TCA cycle happens inside mitochondria, the reduction of intermediates of TCA cycle is probably due to serum starvation-induced mitochondrial damage and oxidative stress. The metabolic impacts of serum starvation are primarily on cellular energy metabolism by modulating the alteration of energy regulating proteins (e.g p-AMPK, p-mTOR, p-ACC)^20^. These metabolic changes were consistent with the transcriptional expression of most genes that are responsible for encoding the catalyzing enzymes in the energy metabolism pathways. As a result, our current observation suggested that LoVo cells may undergo a metabolic shift from glucose- to glutamine-derived energy supply during the period of 96 h serum starvation. Meanwhile, an obvious increase of 3-hydroxybutyric acid was observed in serum-starved cells, which is one of the end-products of aerobic glycolysis and belongs to ketone bodies. Study indicates that administration of 3-hydroxybutyric acid increases tumor growth and stimulates the migration of epithelial cancer cells^47^. Although the role of up-regulated 3-hydroxybutyric acid is not clear, we speculated that the overproduction of 3-hydroxybutyric acid might be a secondary response to increase tumor cell survival upon energy deficiency during serum starvation.

### Summary

Our current study indicated that serum starvation could induce comprehensive alterations both at transcriptional and metabolic levels. The serum starvation-related transcriptional alterations are mainly involved in regulation on cell death, apoptosis, autophagy, cell cycle, DNA replication and p53 signaling pathways, whereas the altered metabolic pathways included TCA cycle, glucose and amino acids metabolism, as well as nucleic acids metabolism. ADMA treatment could restore most of the transcriptional changes induced by serum starvation, but has minor impacts on metabolic changes. It is envisaged that the metabolic changes were likely to be a consequence of serum starvation, but not either promoted or inhibited apoptosis because ADMA could suppressed the serum starvation-induced apoptosis and transcriptional alterations without impacts on cell metabolism. Although we could not draw a conclusion on the mechanisms through which ADMA prevented apoptosis in current report, our results suggested that the anti-apoptosis effect of ADMA was associated with transcriptional modulation on effector caspases, and FAS genes, but independent of cell metabolism. Further studies are warranted for determining the exact roles of these identified genes in modulating the anti-apoptosis effect of ADMA in LoVo cells.

## Additional Information

**How to cite this article**: Zheng, N. *et al*. Effects of ADMA on gene expression and metabolism in serum-starved LoVo cells. *Sci. Rep.* **6**, 25892; doi: 10.1038/srep25892 (2016).

## Supplementary Material

Supplementary Information

Supplementary Table 1

Supplementary Table 2

## Figures and Tables

**Table 1:** The enriched KEGG pathways with serum starvation-regulated genes.

**Pathway**	**KEGG ID**	**P value**
Cell cycle	hsa04110	<0.001
DNA replication	hsa03030	<0.001
Pyrimidine metabolism	hsa00240	<0.001
p53 signaling pathway	hsa04115	<0.001
Spliceosome	hsa03040	<0.001
Base excision repair	hsa03410	<0.001
Nucleotide excision repair	hsa03420	<0.001
Mismatch repair	hsa03430	<0.001
Glutathione metabolism	hsa00480	0.0012
Purine metabolism	hsa00230	0.0015
RNA degradation	hsa03018	0.031

**Table 2:** The differential metabolites among groups.

**Metabolites**	**RT_min**	**Mean** **±** **SD**			**Fold Change**		**P value**	
		**C**	**SS**	**ADMA**	**SS/C**	**ADMA/C**	**SS vs C**	**ADMA vs SS**
Hypotaurine^LC^	3.07	2.060 ± 0.340	1.174 ± 0.157	1.205 ± 0.240	0.570	0.585	0.001	0.815
2,3-Diaminopropionic acid^LC^	3.40	0.921 ± 0.055	0.684 ± 0.073	0.740 ± 0.083	0.742	0.803	0.000	0.290
Aminoadipic acid^LC^	3.54	2.209 ± 0.275	1.159 ± 0.161	1.084 ± 0.133	0.525	0.491	0.000	0.443
5-Aminolevulinic acid^LC^	3.63	0.409 ± 0.125	0.174 ± 0.020	0.169 ± 0.015	0.425	0.412	0.003	0.648
Asparagine^LC^	3.67	0.990 ± 0.196	0.476 ± 0.060	0.447 ± 0.079	0.481	0.451	0.001	0.524
N-Acetyl-D-glucosamine^LC^	3.75	0.092 ± 0.006	0.141 ± 0.013	0.148 ± 0.011	1.532	1.613	0.000	0.360
Glutathione^LC^	3.97	17.440 ± 2.498	0.281 ± 0.039	0.229 ± 0.131	0.016	0.013	0.000	0.416
Deoxyguanosine^LC^	4.03	0.756 ± 0.077	1.079 ± 0.253	0.985 ± 0.200	1.427	1.302	0.026	0.531
Pyruvic acid^GC^	5.37	0.175 ± 0.054	0.091 ± 0.026	0.152 ± 0.041	0.520	0.868	0.014	0.022
3-Hydroxybutyric acid^GC^	6.93	0.024 ± 0.007	0.044 ± 0.010	0.040 ± 0.010	1.827	1.661	0.007	0.548
Urea^GC^	8.00	0.009 ± 0.001	0.017 ± 0.002	0.017 ± 0.001	1.835	1.924	0.000	0.406
Ethanolamine^GC^	8.48	0.043 ± 0.010	0.171 ± 0.018	0.189 ± 0.025	4.002	4.418	0.000	0.235
Proline^GC^	8.92	1.285 ± 0.439	2.946 ± 0.368	2.691 ± 0.545	2.292	2.094	0.000	0.413
Glycine^GC^	9.05	1.426 ± 0.389	3.025 ± 0.581	2.765 ± 0.385	2.121	1.939	0.001	0.429
Succinic acid^GC^	9.06	0.041 ± 0.010	0.027 ± 0.003	0.026 ± 0.003	0.655	0.630	0.014	0.595
Glyceric acid^GC^	9.39	0.026 ± 0.010	0.062 ± 0.005	0.072 ± 0.004	2.426	2.820	0.000	0.006
Uracil^GC^	9.46	0.060 ± 0.006	0.111 ± 0.022	0.129 ± 0.042	1.837	2.151	0.001	0.403
Fumaric acid^GC^	9.50	0.124 ± 0.026	0.025 ± 0.004	0.025 ± 0.005	0.202	0.199	0.000	0.927
Aminomalonic acid^GC^	11.22	0.011 ± 0.007	0.025 ± 0.008	0.023 ± 0.009	2.291	2.095	0.015	0.692
Nicotinamide^GC^	11.23	0.053 ± 0.064	0.164 ± 0.034	0.184 ± 0.045	3.084	3.459	0.009	0.451
Malic acid^GC^	11.44	0.448 ± 0.090	0.071 ± 0.019	0.062 ± 0.007	0.159	0.139	0.000	0.362
Methionine^GC^	11.80	0.089 ± 0.024	0.123 ± 0.011	0.122 ± 0.015	1.390	1.370	0.018	0.846
Aspartic acid^GC^	11.82	1.754 ± 0.454	0.649 ± 0.108	0.616 ± 0.110	0.370	0.351	0.001	0.644
5-oxoproline^GC^	11.86	4.664 ± 2.358	1.091 ± 0.107	0.831 ± 0.472	0.234	0.178	0.010	0.264
4-hydroxy-proline^GC^	11.91	0.052 ± 0.020	0.004 ± 0.001	0.004 ± 0.001	0.075	0.069	0.001	0.620
Threonic acid^GC^	12.20	0.024 ± 0.009	0.006 ± 0.001	0.006 ± 0.000	0.244	0.265	0.002	0.271
2-Hydroxyglutaric acid^GC^	12.47	0.008 ± 0.002	0.004 ± 0.001	0.003 ± 0.000	0.485	0.444	0.004	0.358
Glutamate^GC^	12.99	1.828 ± 0.522	0.193 ± 0.031	0.187 ± 0.033	0.106	0.102	0.000	0.749
Homocysteine^GC^	13.51	0.002 ± 0.000	0.009 ± 0.001	0.008 ± 0.002	5.833	5.234	0.000	0.380
Xylose^GC^	13.81	0.075 ± 0.019	0.208 ± 0.030	0.234 ± 0.019	2.765	3.121	0.000	0.127
Arabinose^GC^	14.15	0.002 ± 0.001	0.005 ± 0.002	0.005 ± 0.001	1.990	2.076	0.014	0.815
Glycerol 3-phosphate^GC^	14.68	0.033 ± 0.006	0.069 ± 0.008	0.069 ± 0.008	2.096	2.096	0.000	0.998
Glutamine^GC^	14.72	0.867 ± 0.147	0.052 ± 0.018	0.044 ± 0.017	0.060	0.051	0.000	0.470
3-Phosphoglyceric acid^GC^	15.17	0.006 ± 0.002	0.034 ± 0.012	0.026 ± 0.007	5.861	4.459	0.001	0.223
Myristic acid^GC^	15.37	0.012 ± 0.002	0.008 ± 0.001	0.009 ± 0.001	0.664	0.709	0.001	0.461
Adenine^GC^	15.71	0.031 ± 0.008	0.016 ± 0.002	0.016 ± 0.003	0.510	0.505	0.004	0.912
Fructose^GC^	15.94	0.018 ± 0.003	0.014 ± 0.001	0.018 ± 0.002	0.756	0.999	0.012	0.001
Mannose^GC^	16.08	0.001 ± 0.000	0.011 ± 0.001	0.011 ± 0.002	7.663	7.346	0.000	0.619
Glucose^GC^	16.21	0.016 ± 0.003	0.048 ± 0.006	0.049 ± 0.012	3.017	3.118	0.000	0.793
Lysine^GC^	16.27	0.135 ± 0.032	0.236 ± 0.026	0.238 ± 0.037	1.756	1.766	0.001	0.945
Pentadecanoic acid^GC^	16.37	0.022 ± 0.002	0.060 ± 0.010	0.067 ± 0.016	2.765	3.103	0.000	0.395
Galactose^GC^	16.40	0.002 ± 0.001	0.008 ± 0.001	0.008 ± 0.002	3.365	3.416	0.000	0.909
Glucuronic acid^GC^	16.63	0.004 ± 0.001	0.013 ± 0.001	0.013 ± 0.002	3.570	3.619	0.000	0.883
Pantothenic acid^GC^	17.03	0.042 ± 0.011	0.011 ± 0.002	0.012 ± 0.001	0.261	0.275	0.000	0.575
Palmitoleic acid^GC^	17.20	0.048 ± 0.008	0.108 ± 0.024	0.125 ± 0.036	2.244	2.608	0.001	0.392
Ribose^GC^	19.10	0.082 ± 0.024	0.033 ± 0.014	0.025 ± 0.016	0.401	0.301	0.004	0.404
Oleic acid^GC^	19.44	0.059 ± 0.008	0.141 ± 0.021	0.159 ± 0.037	2.401	2.708	0.000	0.376
Glucose 1(6)-phosphate^GC^	20.99	0.003 ± 0.001	0.020 ± 0.003	0.012 ± 0.001	7.816	4.661	0.000	0.001
Eicosenoic acid^GC^	21.36	0.004 ± 0.001	0.007 ± 0.001	0.008 ± 0.002	1.833	2.148	0.000	0.166
Orotidine^GC^	21.45	0.007 ± 0.002	0.026 ± 0.011	0.031 ± 0.007	3.986	4.733	0.004	0.420
Myo-Inositol, phosphate^GC^	21.71	0.013 ± 0.002	0.026 ± 0.002	0.031 ± 0.004	1.903	2.335	0.000	0.030
5,8,11,14,17-Eicosapentaenoic acid^GC^	22.41	0.020 ± 0.001	0.027 ± 0.005	0.027 ± 0.005	1.311	1.316	0.017	0.971
Glycerol 1-hexadecanoate^GC^	22.49	0.060 ± 0.004	0.001 ± 0.000	0.001 ± 0.000	0.023	0.021	0.000	0.441
Inosine^GC^	22.52	0.043 ± 0.013	0.197 ± 0.016	0.191 ± 0.018	4.642	4.487	0.000	0.553
Adenosine^GC^	22.91	0.020 ± 0.005	0.048 ± 0.014	0.044 ± 0.017	2.420	2.198	0.003	0.664
Guanosine^GC^	23.87	0.044 ± 0.017	0.098 ± 0.010	0.098 ± 0.026	2.244	2.238	0.000	0.984
Cholesterol^GC^	27.66	2.287 ± 0.295	5.476 ± 0.736	5.715 ± 1.608	2.394	2.499	0.000	0.769

Note: C, SS, and ADMA represent control, 96 h serum starvation and 10 μM ADMA treatment in serum-starved LoVo cells for 96 h, respectively. ^GC^ and ^LC^ means the data are acquired with GC/TOFMS and LC/TOFMS, respectively.
