# Supplementary material for: Effects of ADMA on gene expression and metabolism in serum-starved LoVo cells
Source: Sci Rep. 2016 May 16;6:25892. doi: 10.1038/srep25892 (PMC4867623; doi:10.1038/srep25892)
Supplement: Supplementary Information [file srep25892-s1.pdf]

Title page of supplementary information

Effects of ADMA on gene expression and metabolism in serum-starved LoVo cells

Ningning Zheng, Ke Wang, Jiaojiao He, Yunping Qiu, Guoxiang Xie,

Mingming Su, Wei Jia, Houkai Li

Table S1. The complete enriched GO terms of regulated genes by serum starvation.

Table S2. The ADMA regulated genes at 96 h compared to serum-starved cells

Figure S1. Cluster of differentially expressed genes by ADMA compared to serum starvation using the unsupervised hierarchical clustering method. C represents control group; SS represents 96h serum starvation group; 10μM ADMA for 96h in serum starvation medium.

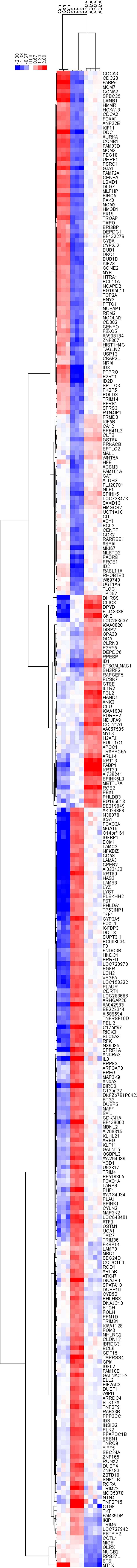
Con
Con
Con
SS
SS
SS
ADMA
ADMA
ADMA
-2.00
-1.33
-0.67
0.00
0.67
1.33
2.00
